# Protein-protein interaction of the putative magnetoreceptor cryptochrome 4 expressed in the avian retina

**DOI:** 10.1038/s41598-020-64429-y

**Published:** 2020-04-30

**Authors:** Haijia Wu, Alexander Scholten, Angelika Einwich, Henrik Mouritsen, Karl-Wilhelm Koch

**Affiliations:** 1 Department of Neuroscience, Division of Biochemistry, University of Oldenburg, D-26111 Oldenburg, Germany; 2 Department of Biology and Environmental Sciences, Neurosensorics/Animal Navigation, University of Oldenburg, D-26111 Oldenburg, Germany; 3 Research Center for Neurosensory Sciences, University of Oldenburg, 26111 Oldenburg, Germany

**Keywords:** Molecular biology, Proteomics, Protein-protein interaction networks

## Abstract

Migratory birds can sense the Earth’s magnetic field and use it for orientation over thousands of kilometres. A light-dependent radical-pair mechanism associated with the visual system is currently discussed as the underlying mechanism of the magnetic compass sense. The blue light receptor cryptochrome 4 (Cry4) is considered as the most likely primary sensory protein that detects the geomagnetic field. Since the protein interaction partners of Cry4 are completely unknown at present, here, we aim to identify potential candidate interaction partners of Cry4 in the avian retina. We used the yeast-two-hybrid system to screen avian cDNA libraries for possible interaction partners of Cry4 in the European robin. The UAS-GAL yeast two hybrid system was applied to confirm a group of candidate Cry4 interaction partners. Six proteins were found to be particularly promising candidates for interacting with European robin Cry4. The identified genes code for guanine nucleotide-binding protein G(t) subunit alpha-2 (GNAT2), long-wavelength-sensitive opsin (LWS, also called iodopsin), guanine nucleotide-binding protein subunit gamma 10 (GNG10), potassium voltage-gated channel subfamily V member 2 (KCNV2), retinol binding protein 1 (RBP1) and retinal G protein-coupled receptor (RGR). All genes are known to be expressed in vertebrate retinae of different species. We conclude by discussing putative signalling pathways that could connect cryptochrome 4 to one or more of these 6 candidates.

## Introduction

Night-migratory songbirds use the Earth’s magnetic field for orientation during long-distance migration^1,2^. Behavioural studies have provided widely accepted experimental evidence that many night-migratory songbirds employ a magnetic compass as well as a magnetic map for orientation^2–4^. These behavioural studies further showed that birds sense the inclination of the geomagnetic field and not its polarity^1,5,6^; that magnetoreception in migratory birds depends on blue light^7,8^, and that magnetic compass information is detected in both eyes^9,10^ and processed in specific parts of the birds´ visual system^11–13^. Schulten *et al*. (1978) suggested in a seminal study^14^ that a light-dependent radical-pair mechanism could be the primary molecular event in birds’ magnetic sense. In a follow-up paper, the class of blue-light receptor proteins called cryptochromes (Cry) was proposed to act as the primary sensor molecule that detects the geomagnetic field^15^. Cry proteins are flavoproteins containing flavin adenine dinucleotide (FAD) as a co-factor and they are known to be involved in circadian clock processes of many organisms^16^. They contain a photolyase-like domain, but do not exhibit photolyase activity. FAD is known to be the molecular component absorbing the light and initiating the photo-chemical reactions that leads to magnetically sensitive radical-pair intermediates^15,17–19^. Recently, the first crystal structure of a bird cryptochrome was solved^20^.

The retinae of both eyes of night-migratory songbirds are considered to be the light-sensitive organs in magnetoreception^9,10^ and subsequent studies located four different Crys (Cry1a, Cry1b, Cry2 and Cry4) in the retinae of night-migratory songbirds. Cry1a from garden warblers (*Sylvia borin*) has been suggested to form long-lived, light-induced radical pairs *in vitro*^21^ and Cry1a of European robins (*Erithacus rubecula*) is, within the retina, exclusively localized in the outer segments of UV sensitive cones^22^. Cry1b is mainly located in ganglion cells of migratory European robins, migratory Northern Wheatears (*Oenanthe oenanthe*), and homing pigeons (*Columba livia*)^23,24^. Cry2 is located in cell nuclei throughout the retina, which is more consistent with a role of Cry2 in the control of the circadian clock than with a role in magnetoreception^25^. Very recently, Günther *et al*.^26^ demonstrated that Cry4 is expressed in the outer segments of the double cone and long-wavelength single cone photoreceptor cells in the retinae of migratory European robins, which could be an ideal location for a primary magnetoreceptor molecule^18^.

Although the signalling state in a Cry molecule from a night-migratory songbird is presently unknown, the current working hypothesis suggests that photoexcitation of the FAD co-factor in Cry followed by electron transfer from three or four neighbouring tryptophan residues leads to the formation of a magnetically sensitive radical pair (summarized in ref. ^18^). The direction of the magnetic field influences, how much of the Cry goes back to the ground state and how much of the Cry is converted to a signalling state. This process probably leads to a conformational change in the C-terminus of Cry4 that is also observed in other Cry variants^18,20,27–29^. This photo-excited Cry would then trigger a magneto-sensitive cell response via protein-protein interactions. Therefore, the protein interaction partners of Cry are expected to be crucial components of a magnetic field sensitive signal transduction pathway, which is completely unknown at present. Furthermore, to act as a compass sensor, Cry would have to be anchored to the cell membrane, e.g. in the outer segments of the photoreceptors, but the anchoring mechanism is also currently unknown.

Qin *et al*.^30^ have reported the results of a computational screening approach based on an alternative hypothesis. They predicted that an avian magnetoreceptor could be an iron-sulphur protein, and they claimed that the ubiquitously present protein IscA1 (dubbed MagR) is a direct interaction partner of Cry4. They furthermore proposed that Cry4 and IscA1 form an iron-containing needle-like protein complex which can work as a ferromagnetic (compass needle like) avian magnetic sensor. Shortly after publication of this work, however, several serious concerns have been raised in the literature^31–35^. These concerns will be addressed in more detail in the discussion part.

The aim of the present study is to *de novo* identify potential Cry interacting proteins in the avian retina focussing primarily on European robin Cry4 (ErCry4). We used the yeast-two-hybrid system (Y2H)^36^ to screen avian cDNA libraries without relying on any preconceived prediction about the nature or characteristics of the ErCry4 interacting proteins.

## Results

### Validation of screening systems

**Figure 1:**
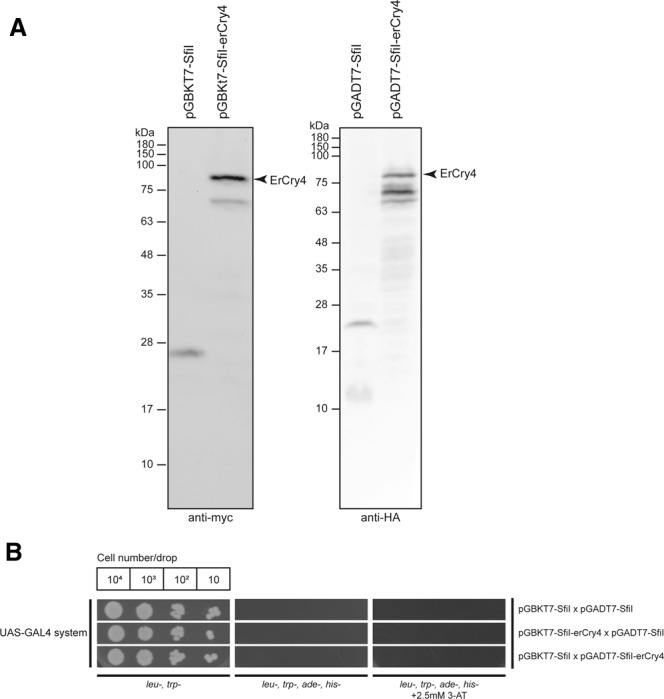
Testing the usability of the UAS-GAL4 Y2H system in screening for ErCry4 interaction partners. (**A**) Western Blots demonstrate the expression of ErCry4 (arrow) using the pGBK7-Sfil (left panel) or the pGADt7-Sfil vector (right panel). No expression was seen in the empty control vector (left lanes in both panels). The HA antibody (1:500) was used to detect pGADT7-SfiI expressed proteins and the myc antibody (1:500) was used to detect pGBKT7 expressed proteins. Different blots were used due to different antibodies as indicated. Full-length blots are provided in the supplement (Figure S3B). (**B**) Serial drop tests were used to verify the background interaction of ErCry4 in the UAS-GAL4 system systems. Yeast cells were mated by two haploid cells containing the respective parts of a pair of the plasmids (for the UAS-GAL4 system) and diluted to the concentration of 10^6^, 10^5^, 10^4^, and 10^3^ cells/ml in sterilized H_2_O and 10 µl of each cell suspension was dropped as a spot. Dropped cells grew on the selective plates lacking leucine, adenine (SD*-leu-trp*), leucine, adenine, tryptophane, histidine (SD*-leu-trp-his-ade*), or leucine, adenine, tryptophane, histidine supplemented with 2.5 mM 3-Amino-1,2,4-triazole (3-AT) for three days at 30°C. Blank parts of plates were cut to facilitate visibility of relevant parts. Full plates are shown in the supplement (Figure S5).

We used the UAS-GAL4 system (Supplementary Figures S1 and S2) for the screening approach. To validate the Y2H systems for this purpose we tested first whether yeast cells transfected separately by each single Y2H vector construct could express the expected bait or prey proteins. Such expression is essential for the Y2H approach to work. Furthermore, we tested for any background signals due to auto-activation by comparing the immunoblot results of empty Y2H vectors with ErCry4 containing vectors (Fig. 1A). The UAS-GAL4 system showed strong expression with both plasmids (bait and prey proteins), although slight degradations were apparent (Fig. 1A).

Further, we used a serial drop test (Fig. 1B) to test for any background interaction of ErCry4 with empty Y2H vectors and thus with any unspecific proteins present in the yeast cells. Cells were diluted to the concentration of 10^6^, 10^5^, 10^4^, and 10^3^ cells/ml in sterilized H_2_O and 10 µl cell suspension was dropped at each spot. Dropped cells grew for three days at 30 °C on selective plates lacking either (1) leucine, tryptophan (SD*-leu-trp*) or (2) leucine, adenine, tryptophan, histidine (SD*-leu-trp-his-ade*) or (3) leucine, adenine, tryptophan, and histidine supplemented with the histidine competitor 3-Amino-1,2,4-triazole. In conclusion, the GAL4 system showed no background interaction of ErCry4 under all tested conditions. Thus, the UAS-GAL4 system turned out to be a valid choice for Y2H screening of ErCry4 interaction partners. In the initial phase of the project, we compared the UAS-GAL4 system with the split-ubiquitin system, which would have been a reasonable choice as it can be applied for membrane associated protein-protein interaction processes (see supplementary information). However, the split-ubiquitin system proved to be less valid and was therefore not applied in our screening efforts. Nevertheless, we supply the initial testing results of the split-ubiquitin system in the supplement (Figure S3).

**Figure 2:**
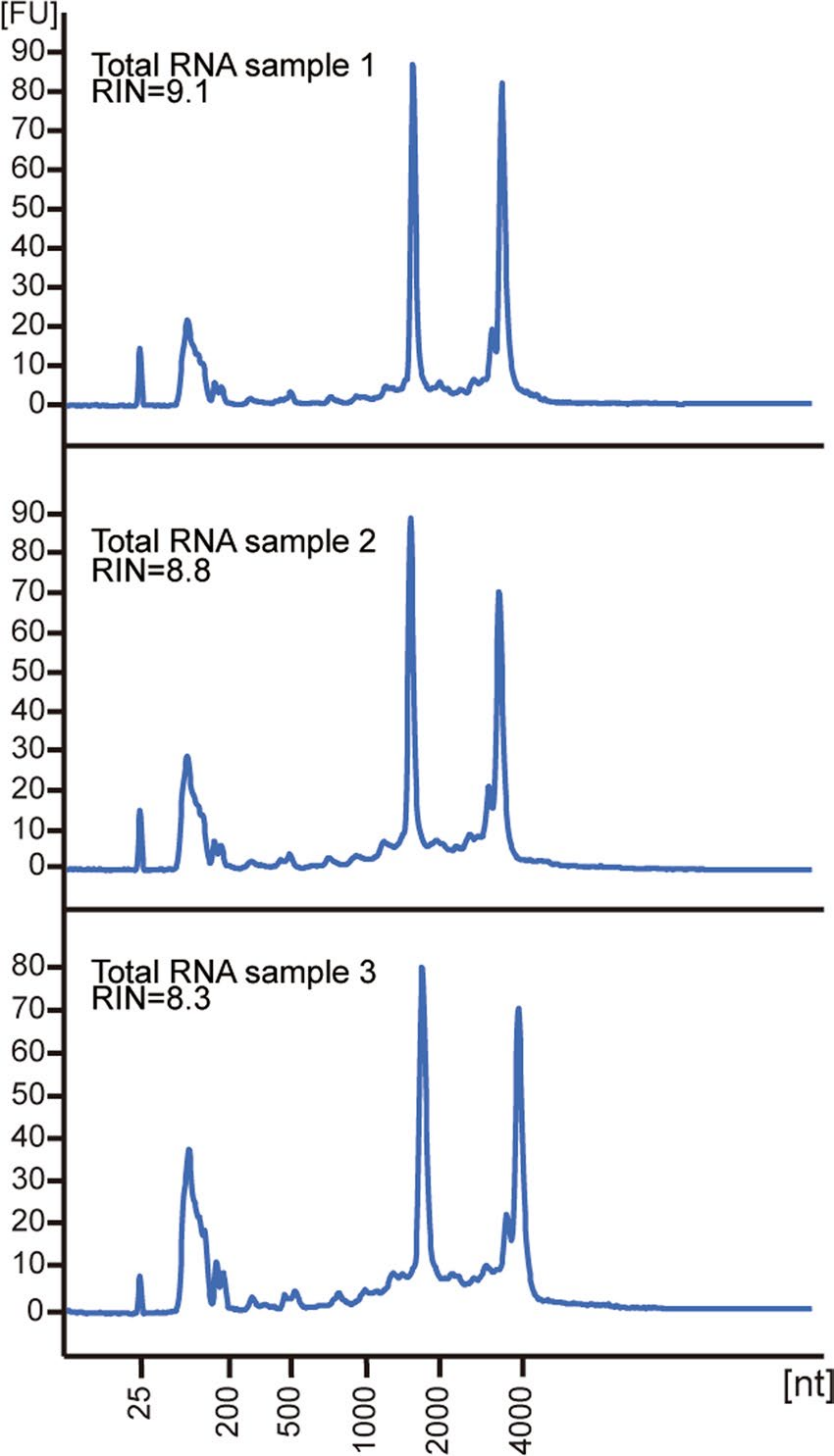
RNA quality test yielding RIN values of 9.1, 8.8 and 8.3. Y-axis: Fluorescence intensity in arbitrary units, x-axis: number of nucleotides.

To ensure a genome wide Y2H screening, cDNA libraries with high quality were created by using total RNA extracted from European robin retina. Three independent batches of RNA from three different individuals were obtained yielding RNA integrity numbers (RIN) of 8.3, 8.8 and 9.1, which indicate intact total RNA of sufficient quality (Fig. 2). Three cDNA libraries were generated from each set of RNA and all nine cDNA libraries were cloned into vectors and used to transform *E.coli* cells for amplification. Subsequently, yeast cells were transformed with these vectors containing the cDNA libraries. To estimate the gene complexity of the cDNA libraries, both *E.coli* and yeast transformants were counted and the approximate coverage rate of the European robin open reading frame (ORF) was calculated (Table 1). We have access to a draft European robin genome (access will be provided through a big consortium paper currently under review) estimating ca. 20,000 genes, which seems to fit with the typical values reported by the avian genome sequencing project^37^. Since each cDNA insert is connected to the N-terminal prey sequence of the vector by 5’UTR (5’ untranslated region) of unknown length, only one third of the cDNAs in the libraries is considered to generate products with a correct ORF. In total, the nine created cDNA libraries cover around 85-times the total number of genes and, therefore, the coverage of the yeast cDNA screening systems should be around 40-times of the total number of genes (see Table 1).

**Table 1:** Gene coverage rate of cDNA libraries.

cDNA library	Colonies generated	ORF coverage rate
*E.coli*	1.7 ± 0.8×10^6^	~8500%
*S.cerevisiae*	8.1 ± 1.3×10^5^	~4050%

### Identification of putative Cry4 interaction partners

Based on the validation tests described above, we applied the UAS-GAL4 system to identify a group of candidate ErCry4 interaction partners. The advantage of the UAS-GAL4 Y2H system is that the prey and the bait vectors can be constructed to harbour binding and activating factors at the N-terminus of the corresponding insert in both plasmids (for example ErCry4 as bait). Furthermore, the stability of ErCry4 protein expression in both vectors was higher in the UAS-GAL system than in the split-ubiquitin system as detected by immunoblotting (Fig. 1 and Figure S3).

**Figure 3:**
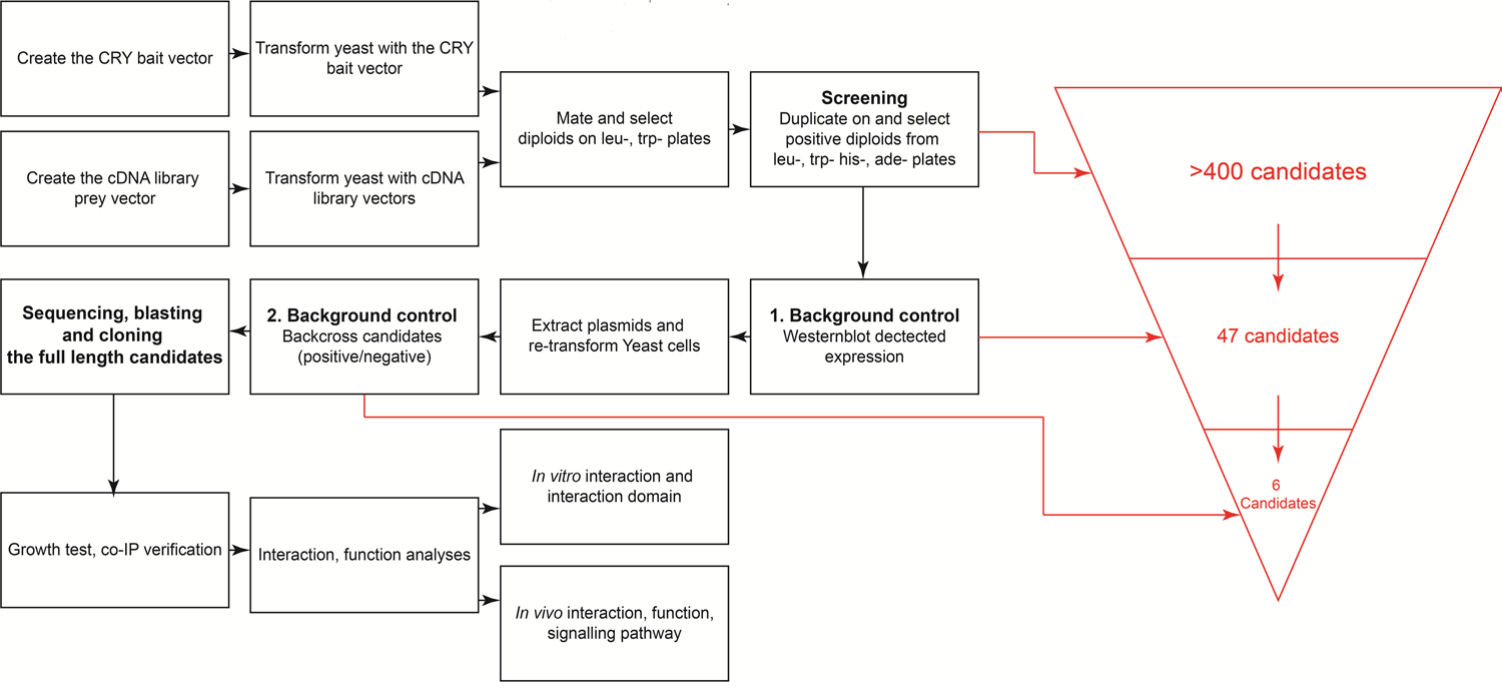
Workflow chart of the screening approach we used for the Y2H system.

An overview of the workflow featuring the steps of transformation, screening, background control and validation is shown in Fig. 3 and will be described in more details below.

**Figure 4:**
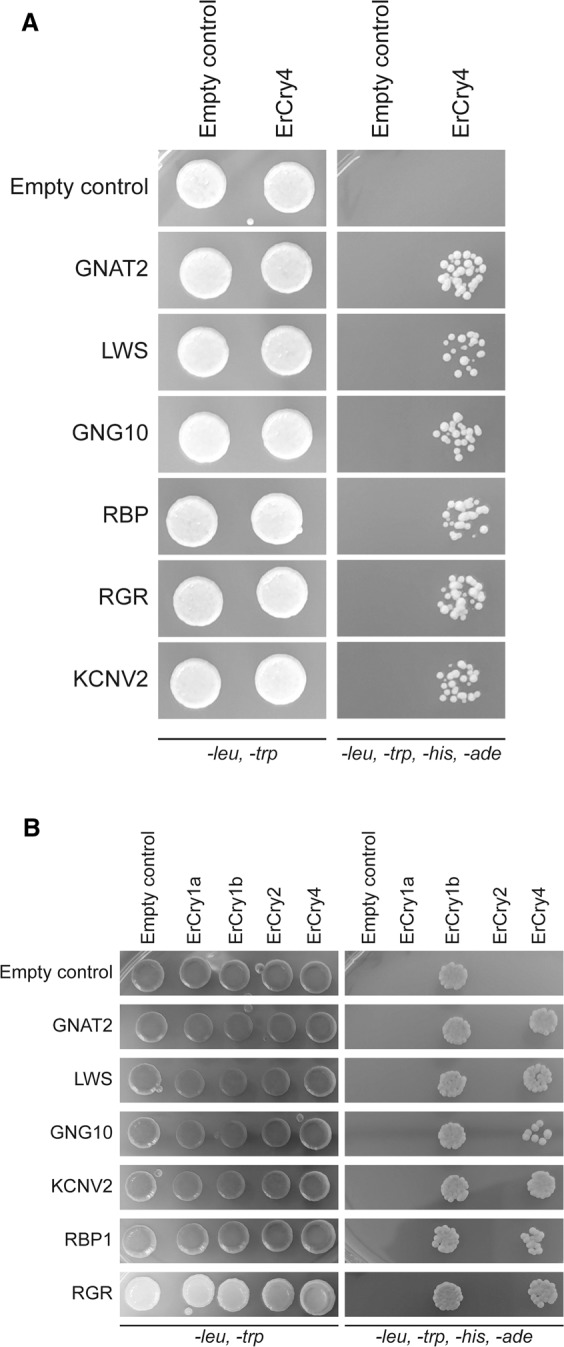
Testing of the six candidates selected as potential interaction partners of ErCry4. (**A**) Full length genes of the candidates were cloned into the pGADT7-SfiI vector. Left: All yeast strains grew on the control plates as expected. Right: All the six full-length candidates interacted with ErCry4 but not with the empty vectors or when grown on plates lacking leucine, histidine, tryptophan and adenine, demonstrating a specific interaction (the two vectors support growing of yeast cells on plates lacking leucine and histidine due to their selective markers). A positive interaction triggers the reporter gene expression leading to a growth on the plates lacking tryptophan and adenine. Different plates are displayed based on the candidate genes that were selected from more than 400, which were found at early stages of the screening efforts (see main text). Full plates are shown in Figure S4A. (**B**) Testing for putative interactions between various Cry forms and putative interaction partners of ErCry4. Specific information about plasmids and yeast strains is given in the Methods section. Yeast strains containing the corresponding plasmids mated and 10^4^ of the formed diploid cells were dropped on to SD-leu-trp and SD-leu-trp-his-ade plates. pGBKT7-SfiI and pGADT7-SfiI empty plasmids were used as negative controls. Although interactions were observed only between ErCry1b and ErCry4 with the putative interaction partners, there is also an interaction visible between ErCry1b and the empty control vector. Rows of signals were cut and assembled allowing an overview of the interaction pattern of Cry variants. Full-size plates are shown in Figure S4B.

While false positive results are common in Y2H screening, they are, however, reducible. We followed several steps to reduce the risk of potential false positive results. If 3′UTR and 5′UTR are included in the cDNA structure, the total length of these two non-ORF elements could reach a large size. Therefore, candidates smaller than 1 kb will not be considered and RNAs smaller than 1 kb were removed from the total RNA. Further, if the expression products are only 10 kDa of length, they most likely come from the 3’UTR with a pre-mature termination due to an early stop codon originating from a frame-shift reading of the UTR. Thus, polypeptides smaller than 10 kDa were not considered further. Finally, any self-activation of potential candidates which leads to signals with an empty vector was removed from the candidate list. Expression of putative candidates was verified through immunoblotting. Indeed, a large number of candidates (more than 400) showed up at early stages of the Y2H procedure. However, applying these rigorous selection procedures, we narrowed down the number of potential candidates to six proteins or protein subunits (Fig. 3) which appeared to interact highly specifically with ErCry4. Representative results are shown in Fig. 4 in form of a drop test. Cells were diluted to the concentration of 10^6^ cells/ml in sterilized H_2_O and 10 µl cell suspension was used for each drop. The dropped plates (SD*-leu-trp*/SD*-leu-trp-his-ade*) were further incubated for 3 days at 30 °C. After collecting positive bacteria colonies, their plasmids were isolated and sent in for sequencing. The obtained sequences were blasted against the NCBI databases. This process revealed the phylogenetic homology of the identified candidate genes to other genes of closely related bird species. The identified potential ErCry4 interaction partner genes code for the following proteins (Table 2): guanine nucleotide-binding protein G(t) subunit alpha-2 (GNAT2), long-wavelength-sensitive opsin (LWS, also called iodopsin), guanine nucleotide-binding protein subunit gamma 10 (GNG10), potassium voltage-gated channel subfamily V member 2 (KCNV2), retinol binding protein 1 (RBP1), and retinal G protein coupled receptor (RGR).

**Table 2:** List of potential ErCry4 interaction candidates resulting from a current screening.

Candidate	Most similar ORF	Species	Identity	Gene	NCBI Reference
1	GNAT2	Ficedula albicollis	97.18%	G protein subunit alpha transduction 2	XM_005059369.1
2	LWS	Taeniopygia guttata	93.63%	long wavelength sensitive opsin	NM_001076702.1
3	GNG10	Numida meleagris	98.07%	G protein subunit gamma 10	XM_021379609.1
4	RBP1	Sturnus vulgaris	97.79%	retinol binding protein 1	XM_014870256.1
5	RGR	Ficedula albicollis	96.28%	retinal G protein coupled receptor	XM_005048136.2
6	KCNV2	Ficedula albicollis	98.72%	Potassium voltage-gated channel subfamily V member 2	XP_016160160.1

To test whether the identified Cry4 interaction partner candidates also interact with any of the other cryptochromes known to be expressed in the European robin retina, we tested whether ErCry1a, ErCry1b and ErCry2 interacted with each of these six candidates. Drop tests as described above and like the one shown in Fig. 4A revealed no specific interaction of ErCry1a and ErCry2 with any of the six candidates (Fig. 4B). Interaction of ErCry1b was inconclusive as it showed an interaction with all six candidates, but also with the empty control vector, suggesting a general unspecific interaction generated by ErCry1b itself. This suggests that ErCry4 differs from the other Cry forms in European robin, and that ErCry4 interacts with its own set of specific interaction partners.

**Figure 5:**
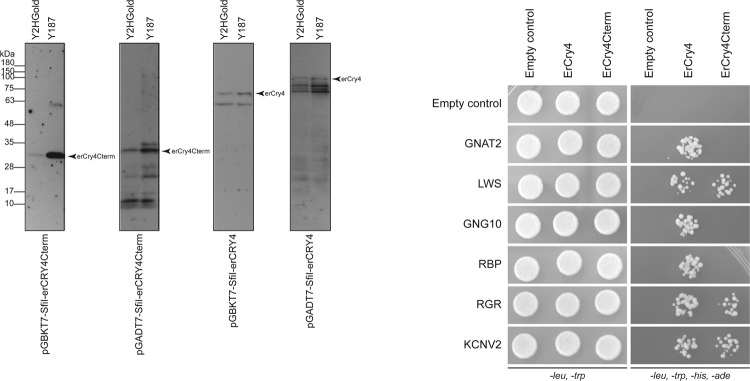
Western blots detected the expression of full length ErCry4 and the C-terminal fragment only of ErCry4 (ErCry4Cterm) in different Y2H strains (left panel). Full length ErCry4 and ErCry4Cterm were cloned into both Y2H plasmids. All four plasmids were used to transform both Y2H strains, respectively. Proteins were detected by antibodies against their tag; anti-myc and anti-HA were used to detect proteins expressed from plasmids pGBKT7 and pGADT7, respectively. Blot was cut and assembled. The full-size blot is provided in the supplement (Figure S6). Drop tests (right panel) were used to probe the interaction between full length ErCry4 or ErCry4Cterm with the six potential interaction partners, which were identified from the Y2H screening. Cells were diluted to a concentration of 10^6^ cells/ml in sterilized H_2_O and 10 µl cell suspension was used for each drop. Full-size plates are provided in the supplement (Figure S7).

Based on structural analyses suggesting that the C-terminal region of bird Cry4 is predicted to be a signalling domain^20,27,38^, we reasoned that the C-terminal part of ErCry4 (ErCry4Cterm, consisting of 180 nucleotides or 60 amino acids) might constitute a specific target region for potential interaction partners. Therefore, we cloned the last 60 C-terminal amino acids into the UAS-GAL Y2H system. By this approach, we improved the screening efficiency for potential signalling cascade interaction partner candidates. Expression of the C-terminus of ErCry4 is stable and strong, especially in combination with the bait vector pGBKT7-SfiI and the Y187 yeast strain (Fig. 5, left panel). We then tested which of the six candidates interacts with the C-terminus of ErCry4 using the same drop test as in Fig. 4. We identified three out of our six candidates to specifically interact with the C-terminus of ErCry4. These were the genes coding for long wavelength opsin (LWS), retinal G protein-coupled receptor (RGR) and potassium voltage-gated channel subfamily V member 2 (KCNV2) (Fig. 5, right panel). Furthermore, we tested whether ErCry4 expression is specifically affected by blue light, but so far, we have no evidence for that (Figure S6). It is important to stress that even though blue light most likely plays an important role in the mechanism *in vivo*, there can be many good reasons why Y2H screening results would nevertheless not be light-dependent.

### Interaction of ErCry4 with IscA1 and clock proteins

**Figure 6:**
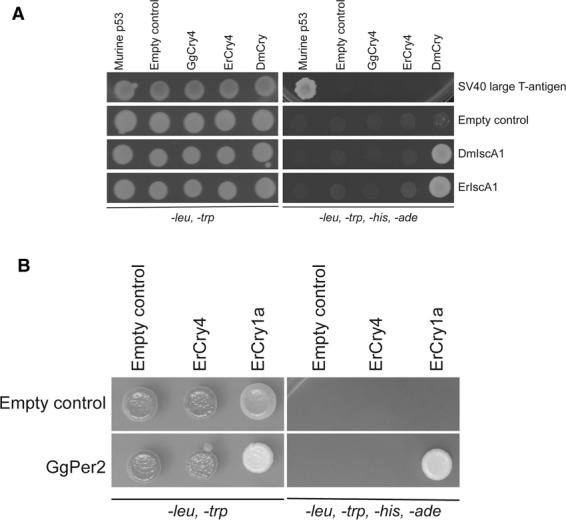
(**A**) Testing for putative interactions between Cry and IscA1 variants. Specific information about plasmids and yeast strains is given in the Methods section; pGBKT7-Murine P53 and pGADT7-SV large T-antigen were used as positive control and pGBKT7-SfiI and pGADT7-SfiI empty plasmids were used as negative control. (**B**) Testing for interaction between Per2 and ErCrys. Corresponding yeast strains mated and 10^4^ of the formed diploid cells were dropped on to SD*-leu-trp* and SD*-leu-trp-his-ade* plates. pGBKT7-SfiI and pGADT7-SfiI empty plasmids were used as negative controls. Interaction was only observed between GgPer2 and ErCry1a. Drop tests were performed on different plates specific for the indicated candidates. Rows of signals were cut and assembled allowing an overview of the interaction pattern of Cry variants. Full-size plates are provided in the supplement (Figures S8 and S9).

As mentioned in the introduction, the iron-sulphur protein IscA1 has been suggested to interact with pigeon Cry4^30^. However, whether this interaction also exists in European robin is yet unknown. Using our Y2H system, we tested whether different Cry proteins and IscA1 proteins can form a complex (Fig. 6A). In addition to ErCry4, full-length genes of Cry of *Drosophila melanogaster* (DmCry), Cry4 of *Gallus gallus* (GgCry4), IscA1 of *Erithacus rubecula* (ErIscA1) and IscA1 of *Drosophila melanogaster* (DmIscA1) were cloned into a Y2H construct. Putative interactions between Cry and IscA1 variants was probed by a drop-test as described above. We found no evidence for an interaction between ErCry4 and ErIscA1 (Fig. 6A). Furthermore, GgCry4 neither interacted with ErIscA1 nor with DmIscA1 (Fig. 6A). Drop-tests in Fig. 6A only indicated an interaction between DmCry and IscA1 from drosophila and European robin. The binding of murine p53 with the SV40 large T-antigen served as a positive control.

Moreover, circadian and seasonal expression profiles of ErCry isoforms showed that ErCry4 expression is not circadian whereas the expression of Cry1a, Cry1b and Cry2 is strongly circadian^26^. To test more directly whether ErCry1a and ErCry4 could be involved in the circadian clock of the birds, we used the Y2H system to probe the interaction between the clock protein known to interact with Cry in many clocks (period protein 2, Per2) and ErCry1a or ErCry4, respectively. For this purpose, the full-length Per2 gene of *Gallus gallus* (GgPer2) was cloned into the Y2H construct. We tried by several independent approaches without success to clone the Per2 gene of European robin using the sequence information provided by the draft genome of the European robin (see above). Therefore, the GgPer2 construct served as a second-best substitute in the test. We nevertheless found a clear interaction between GgPer2 and ErCry1a, but no interaction between GgPer2 and ErCry4 (Fig. 6B). These findings strongly support our previous conclusion^26^ that ErCry1a but not ErCry4 might be involved in regulation of circadian rhythms in night-migratory songbirds.

## Discussion

Sensing of the earth´s magnetic field requires a primary receptor molecule that interacts with signalling proteins. These interactions result in a cell response, which only then can be processed by the retina and the brain of the organism (e.g. a navigating bird). As outlined in the introduction, we currently consider ErCry4 to be the most likely putative magnetoreceptor candidate because of what is currently known about its expression, cellular localization, and photochemistry^26,39^. Here, we identified six proteins consistently interacting with ErCry4. It became immediately apparent that four of the six candidates represent proteins that typically operate in signalling pathways. The other two, the retinol binding protein and the retinal G protein coupled receptor, are involved in the transport and isomerization of the chromophore all-*trans*-retinal. Therefore, we considered the following four of the identified six candidate proteins most likely to be potentially involved in a magnetosensory signalling pathway: guanine nucleotide-binding protein G(t) subunit alpha-2 (GNAT2), long-wavelength-sensitive opsin (LWS), guanine nucleotide-binding protein subunit gamma 10 (GNG10), and potassium voltage-gated channel subfamily V member 2 (KCNV2).

**Figure 7:**
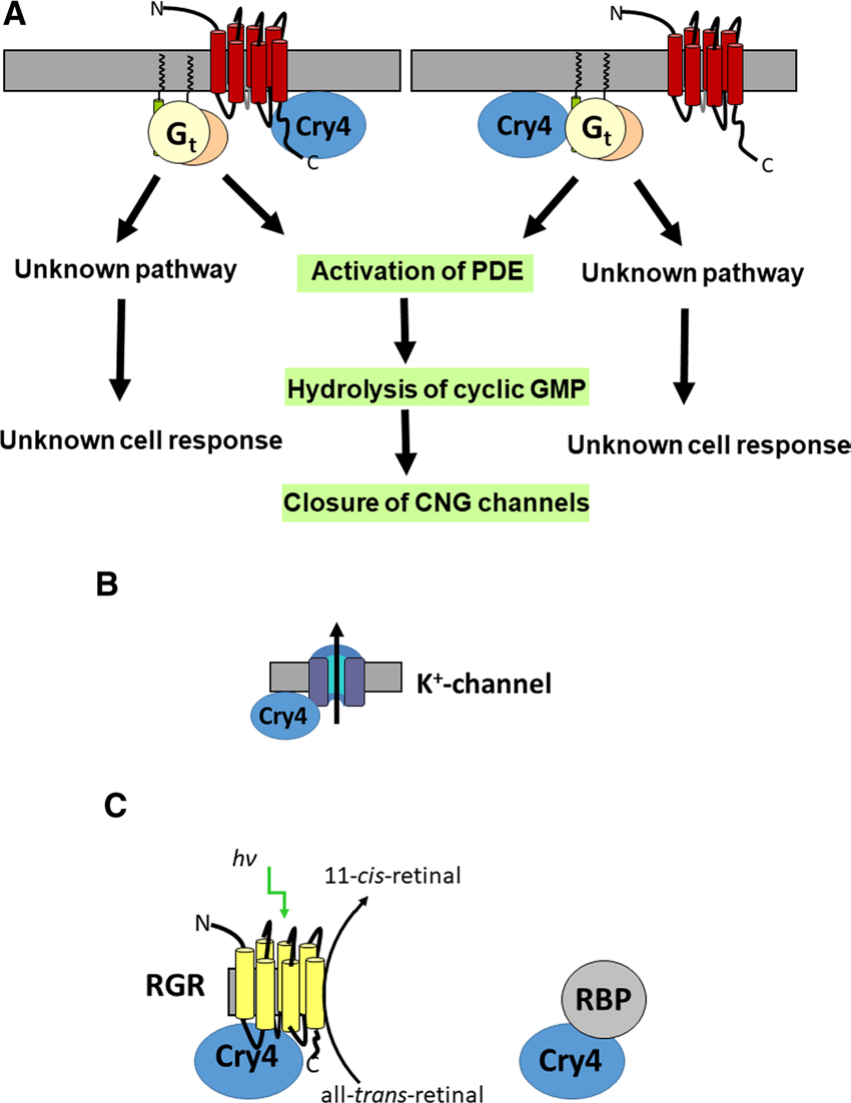
Potential signalling pathways in magnetoreception involving ErCry4 and its hypothetical interaction partners. (**A**, left scheme) ErCry4 might form a complex with long-wavelength-sensitive opsin triggering activation of G protein (G_t_α)-mediated phototransduction. Alternatively, interactions between ErCry4 and G_t_α could also lead to activation of downstream signalling proteins not involving opsin (A, right scheme). The downstream events could be part of the classical phototransduction pathway leading to closure of cyclic nucleotide-gated (CNG) channels via hydrolysis of cGMP. However, an unknown pathway leading to an unknown cell response could be involved. Both G_t_α and G_t_γ are attached to membranes by lipid anchors. Thus, they might anchor ErCry4 to the surface of the disk membrane. The seven-transmembrane receptor opsin could also serve as a membrane anchor for ErCry4. (**B**) A potential complex involving ErCry4 and the voltage-gated potassium channel subunit K_v_8.2, which is the modifying subunit in a heteromeric K^+^-channel, could directly affect the membrane potential of the cell. (**C**) Putative interaction of ErCry4 with RBP and the retinal photoisomerase RGR that catalyzes the transformation of all-*trans*-retinal to 11-*cis*-retinal.

Long wavelength-sensitive opsin (also called red opsin or iodopsin) is the photoreceptor molecule present in the long wavelength sensitive single cones and in the double cones. Noteworthy, the interaction of ErCry4 with red opsin matches the localization of ErCry4 in long wavelength single cones and double cones of night-migratory European robins that Günther *et al*. demonstrated in ref. ^26^. They further showed that robin Cry4 does not co-localize with other opsin forms. Our screening results support these findings, since we did not find any interactions between ErCry4 and any opsin forms other than LWS. Two obvious putative interpretations of an interaction between ErCry4 and red opsin presents themselves: ErCry4 might use red opsin, which is by far the most common membrane protein in the outer segments of double cones and long-wavelength single cones, as an anchor to achieve the restriction in rotation needed for ErCry4 to act as a compass sensor^40–42^. Alternatively, ErCry4 could piggyback on the red opsin signalling cascade which is inactive at night under low-light conditions. Photoexcitation by dim blue light at night would not activate red opsin by photoisomerization of 11-*cis*-retinal. Therefore, there would be no competition with normal vision at night. Thus, the photochemistry of FAD in ErCry4 could trigger a conformational change that would lead to subsequent protein-protein interaction in the otherwise dormant long-wavelength-sensitive opsin signalling cascade (see “conclusions” and Fig. 7 below for further discussion of this option).

Two subunits of the heterotrimeric cone G protein transducin (genes GNAT2 and GNAG10 coding for G_t_α and G_t_γ) were also identified as putative interaction partners of ErCry4 in our screening, but no interaction of ErCry4 with the β-subunit G_t_β was observed. Activation of heterotrimeric G proteins by G protein-coupled receptors normally leads to splitting the G protein complex in Gα and Gβγ, which then pass the signal on to different targets. G_t_α and G_t_γ are fatty-acylated at their N- and C-termini, respectively, providing attachment to the membrane^43^. By this modification, G_t_α and G_t_γ could be putative membrane anchors for ErCry4, which might be the reason why we have no indication of ErCry4 G_t_β interacting with the non-anchoring G_t_β subunit.

A further candidate gene listed in Table 2 is KCNV2 coding for the voltage-gated potassium channel K_v_8.2, which is the modifying subunit in heteromeric delayed rectifier potassium channels forming complexes with the K_v_2 channel family subunits^44^. K_v_8.2 is expressed in the primate and mouse retina showing a highly compartmentalized localization in inner segment membranes^45,46^. Mutations in the KCNV2 gene in humans cause cone dystrophies indicating the essential role of K_v_8.2 for normal vision^47^. However, to our knowledge, the expression of avian-specific orthologues of K_v_8.2 is completely unknown and has not been investigated so far. An interaction between ErCry4 and KCNV2 could mean that ErCry4 directly regulates the channel and thus the cone’s membrane potential.

The final two proteins suggested to interact with ErCry4 were previously described as components of the visual cycle^48^. Retinol binding protein (RBP) is a carrier protein that takes up retinol (for example from the liver) and transports it in the bloodstream to different targets. RBP is an essential component of the vitamin A metabolism and immunoreactivity of RBP is widely distributed in the rat eye^49^. The retinal G protein-coupled receptor (RGR) is a non-visual opsin exhibiting photoisomerase activity that can transform all-*trans*-retinal to 11-*cis*-retinal. It is present in the retinal pigment epithelial and Müller glial cells^50,51^. Recently, it was also shown to be expressed in photoreceptors and intrinsically photosensitive ganglion cells of the chicken retina^52^ and in long-wavelength-sensitive and short-wavelength-sensitive cone photoreceptors and some retinal ganglion cells in the human and bovine retina^53^. An involvement of ErCry4 in the visual cycle seems odd at present. However, if such an interaction would materialize, the interaction of ErCry4 with RGR might indicate a linkage of ErCry4 to cone pigment regeneration via supply of 11-*cis*-retinal (Fig. 7C).

Our findings do not support a complex formation of ErCry4 with IscA1 (or MagR), at least not in the European robin retina. This strongly suggests that the MagR hypothesis of Qin *et al*.^30^ cannot explain magnetoreception in European robins. Previously, it was pointed out that the claims made by Qin *et al*.^30^ are inconsistent with basic physical laws^31,32^ and that the measured magnetization curves do not show ferromagnetism at room temperature^32^. Furthermore, Qin *et al*.^30^ reported a widespread distribution of IscA1 and Cry4 in the pigeon retina, which would be untypical for a specialized function^26,32,33^. Although computational modelling supports interaction of drosophila Cry with monomeric IscA1, which we also observed in our drop test (Fig. 6A), formation of elongated IscA1 polymers is unlikely to occur^34^. Recent computational structural work employing molecular dynamics simulation concluded that a direct interaction in ErCry4 and IscA1 could occur with different docking modes^35^. However, the binding process appeared rather weak due to the low number of low binding energy configurations that the authors characterized in the study. They further estimated the distances between the possible molecular components participating in electron transfer reactions (FAD in ErCry4 and iron-sulfur cluster in IscA1, respectively) and could exclude a role of this complex in magnetoreception.

## Conclusions

The identity of the candidate ErCry4 interacting proteins we did identify allows us to speculate about three different putative signalling pathways that appear reasonable based on what is known about the interacting candidates in other species or cell systems (Fig. 7). First, interaction with LWS opsin perfectly matches the previous co-localization studies of Günther *et al*. (2018) showing the presence of LWS opsin and ErCry4 in cone cells (see also introduction)^26^. The interaction with LWS opsin seems to occur via the C-terminus of ErCry4, which might indicate that the interaction is triggered by a conformational change initiated by blue light involving the C-terminus of ErCry4^27^. Thus, ErCry4 could piggyback on LWS cone opsin making use of its downstream phototransduction signalling cascade without the need for direct photo-activation of cone opsin due to the different absorbance characteristics of ErCry4 and LWS opsin^18^. This pathway could include the heterotrimeric G protein complex (G_t_α and G_t_βγ) of the phototransduction cascade, or the interaction of ErCry4 and the G protein could operate independently of the cone opsin pathway triggering unidentified signalling molecules that do not directly interact with ErCry4. Alternatively, our data might indicate that ErCry4 could directly bind to the identified K_v_8.2 channel subunit and control potassium channel function leading to a direct change in the membrane potential. We emphasize that the schemes presented in Fig. 7 are speculative and aim to provide a reasonable interpretation of how the identified interaction partners of ErCry4 could potentially be involved in the molecular and cellular basis of magnetoreception in night-migratory birds. We would like to stress that since ErCry4 mRNA is ubiquitously expressed in European robin tissues (Einwich *et al*. in preparation/personal communication), it must have many different functions other than magnetoreception, and several of the putative interaction partners identified here could be involved in some of these functions. To prove or disprove that one or more of the four (or six) candidates are key components in magnetoreceptive signalling, different experimental approaches in the future will require biophysical/biochemical protein-protein interaction studies *in vitro* and in cellular systems and will finally demand specific gene targeting in birds. It will most likely require many years of additional research to get that far.

## Methods

### Design principles of the study

The classical UAS-GAL4 system (supplementary Figure S1A, upper panel) is based on two split domains (binding domain/BD and activation domain/AD) of a transcription factor termed GAL4 that consists of a binding domain and an activation domain^54^. One domain is fused to the bait (ErCry4 in our case) and the other to putative preys presented in a cDNA library. Interaction of bait and prey causes reassembly of the transcription vector and expression of a downstream reporter gene. A detailed comparison of the results obtained with the UAS-GAL4 system and equivalent non-successful pilot experiments using the split-ubiquitin Y2H-system^54,55^ is provided in the supplementary materials and corresponding figures (Figure S1A),

### Preparation of RNA samples

RNA was extracted from the retina of three European robins which were wild-caught in the vicinity of the university campus using mist nets. The three birds were sacrificed at three different time points (04:00, 13:00, and at 19:00 CET, respectively). All animal procedures were performed in accordance with local and national guidelines for the use of animals in research and were approved by the governmental authorities (Niedersächsisches Landesamt für Verbraucherschutz und Lebensmittelsicherheit/LAVES, Oldenburg, Germany, Az.: 33.12-42502-04-10/0423). All animals were sacrificed by decapitation. Eyes were removed immediately and the retina, free of vitreous, was put into ice-cold TRIzol Reagent (Life Technologies, Carlsbad, CA, USA), shock-frozen in liquid nitrogen and stored at −80 °C until RNA extraction. Total RNA was isolated using TRIzol Reagent (Invitrogen) following the instructions of the manufacturer. RNA concentration was measured using the Infinite 200 PRO instrument (Tecan, Trading AG, Männedorf, Switzerland). RNA quality was determined with the Agilent RNA 6000 Nano Kit using a 2100 Bioanalyzer Instrument (Agilent Technologies, Santa Clara, CA, USA).

### Y2H constructs

In this manuscript, both bait and prey plasmids from the UAS-GAL4 system were modified in a manner that both, the UAS-GAL4 system and the split-ubiquitin system, share the same cloning pattern. The following two oligos were designed:

5′-*TATG***GGGGCCATTACGGCCCGGGAAAAAACATGTCGGCCGCCTCGGCC***G*-3′

5′-*GATCC***GGCCGAGGCGGCCGACATGTTTTTTCCCGGGCCGTAATGGCCCC*C****A*-3′

The bold parts are the same cloning pattern from pPR3N of the split-ubiquitin system (SfiI site underlined) and the italic parts are compatible BamHI/NdeI restriction ends. The oligos were paired by denaturing at 95 °C and re-annealing with a temperature dropping speed of −0.1 °C/second. The double-stranded oligos were 5′-phosphorylated by using T4 PNK and inserted into BamHI/NdeI (New England Biolab, Massachusetts, United States) digested pGBKT7 and pGADT7 plasmids (gifts from Prof. Heike Krebber, University of Göttingen, Germany) and renamed as pGBKT7-SfiI and pGADT7-SfiI, respectively.

All genes were amplified from the cDNA libraries except DmCry and DmIscA1, which are amplified from GH16672 and AT26381 obtained from the Drosophila Genomics Resource Center (DGRC). ErCry4 was amplified and SfiI-digested and cloned into pDHB1 by using primer pair 227/228 and pPR3N, pGBKT7-SfiI, pGADT7-SfiI by using primer pair 227/639. ErCry4Cterm was amplified and SfiI-digested and cloned into pGBKT7-SfiI, pGADT7-SfiI by using primer pair 465/639. GgCry4 was amplified and SfiI-digested and cloned into pDHB1 by using primer pair 225/226 and pPR3N, pGBKT7-SfiI, pGADT7-SfiI by using primer pair 369/226. DmCry was amplified and SfiI-digested and cloned into pDHB1 by using primer pair 502/503 and pPR3N, pGBKT7-SfiI, pGADT7-SfiI by using primer pair 502/504. DmIscA1 was amplified and SfiI-digested and cloned into pDHB1 by using primer pair 499/501 and pPR3N, pGBKT7-SfiI, pGADT7-SfiI by using primer pair 499/500. ErIscA1 was amplified and SfiI-digested and cloned into pPR3N, pGBKT7-SfiI, pGADT7-SfiI by using primer pair 271/272. All primers for cloning full length open reading frames (ORFs) of the candidates are listed in the supplement (Table S2).

### Construction of cDNA libraries

The construction of the cDNA libraries was achieved by using EasyClone cDNA library construction kits (Dualsystems Biotech AG, Schlieren, Switzerland) with some modifications. To synthesize the first strand of cDNA, 2 µg of the total retinal RNAs were reversely transcribed by using CDS-3M primer and the resulting cDNAs were 5′-elogated by using plugOligo 3 M adaptors. These first strand cDNAs were stored at −80 °C.

The first strand cDNA mentioned above was used as template for a PCR reaction by using either DNA polymerase from the kits or KOD DNA polymerase (Merck KGaA, Darmstadt, Germany) following the instructions of the manufacturer. The PCR cycle was set to 21 cycles if using the DNA polymerase from the kits or 25 cycles, if KOD DNA polymerase was used. The PCR products were extracted by using Gel/PCR extraction kits (Nippon Genetics, Japan) and further digested by SfiI (New England Biolab, Massachusetts, United States) and ligated into SfiI digested pGBKT7-Sfi or pGADT7-SfiI plasmids with the molar ratio of 1:1 by using a high concentrated T4 ligase (400 u/µl, New England Biolab, Massachusetts, United States). All ligation reaction was used to transform ultra-high competent *E.coli* cells XL1-gold (Agilent, Santa Clara, U.S.). The transformation reaction were diluted 1:10^3^ and 1:10^6^ for counting the amount of colonies. Transformed cells were collected in 10 ml H_2_O and aliquoted to 1 ml/tube and stored at −80 °C. The aliquot was used for a maxi-plasmid-preparation and the cDNA libraries were acquired. Vector maps are shown in Figure S2.

### Yeast transformation of the cDNA libraries

A single yeast colony (Y2Hgold or Y187) was inoculated in 10 ml yeast extract peptone dextran media (YPD) media as pre-culture. The 10 ml pre-culture was further incubated in 100 ml YPD media for overnight. The OD_546_ of the culture was measured and 30 mL of the cells were collected corresponding to an OD_546_ = 1. The cells were resuspended with 10 ml twice the concentration (2×) of YPD supplemented with adenine (YPAD) and the falcons were washed with 40 ml 2×YPAD. 150 ml 2×YPAD were added to the suspension and the OD_546_ was measured (should be around 0.15). The cells grew with 220 rpm shaking till the OD_546_ reached 0.6 at 30 °C (2x cell divisions, 3–5 hours). The 200 ml culture was divided into four 50 ml falcon tubes and the cells were collected by centrifugation at 700×g for 5 minutes. The cells were washed with 1 ml H_2_O and 1 ml Lithium acetate/Tris-EDTA buffer (LiOAc/TE), respectively, followed by resuspending cells in 600ul LiOAc/TE. Each portion (4×) was set up with: 7 µg library, 100 µl ssDNA (2 mg/ml), 600 µl cells, 2.5 ml PEG/LiOAc. The mixture was vortexed for 1 min and incubated for 45 minutes at 30 °C (short vortexing every 15 minutes). Afterwards, 150 µl DMSO was added and mixed by shaking. The mixture was heat-shocked at 42 °C for 20 minutes. The cells were collected and resuspended in 3 ml YPD and incubated for 90 minutes at 30 °C (shaking at 150 rpm). Finally, the cells were resuspended in 4.8 ml 0.9% NaCl and incubated on selective plates (300 µ l per 150 mm plates).

Meanwhile, the cells were diluted to 1:10^2^, 1:10^3^ and 1:10^4^ and incubated on selective plates for counting efficiency of the transformation. All selective plates for transformation were incubated at 30 °C for 2–3 days and all colonies were washed away from the plates and collected by using 10 ml sterilized H_2_O. The cells were aliquoted to 1 ml/tube and stored at −80 °C.

### Yeast-two-hybrid screening

A single bait colony (*e.g*. Y168 strain with pGBKt7-SfiI-ErCry4 plasmid) was incubated in 50 ml selective medium until OD_546_ reached 0.8. The cells were collected and resuspended into 1 ml selective medium with a concentration of >1 × 10^8^ cells/ml (dilute 1 µl from 1 ml yeast cells 1:1000/1:10000 in 1 ml H_2_O and pour 100 µl on selective plate for controlling the cell amount). One ml of the cDNA library yeast cells was thawed (dilute 1 µl from 1 ml yeast cells with cDNA library 1:1000/1:10000 in 1 ml H_2_O and pour 100 µl on selective plate for controlling the cell amount) and combined with the bait cells. The combined cells were washed twice with 2XYPAD and resuspended in 45 ml 2XYPDA (containing 50 ug/ml kanamycin). The cells were incubated in a 2 L flask at 30 °C for 20–24 hours with slow shaking (30–50 rpm) for mating. The cells were collected by centrifugation at 1000 × g for 5 minutes. The flask was washed twice with 50 ml 2XYPDA and the cells were collected. All cells were resuspended in 5 ml H_2_O. One µl of those cells were used in a dilution of 1:10^2^/1:10^4^/1:10^6^ in 1 ml H_2_O and 100 µl of diluted cells was poured on SD/*-leu*, SD/*- trp*, SD/*-leu-trp* and SD/*-leu-trp-his-ade* plates, respectively, for controlling the mating and interaction efficiency. Afterwards, each 200 µl mating cells were poured and incubated on one SD/*-leu-trp* plate at 30 °C for 3 days. Subsequently, all plates were duplicated onto SD/*-leu-trp-his-ade* plates. The SD/*-leu-trp-his-ade* plates were incubated at 30 °C until the colonies grew up.

The colonies, which grew on the SD/*-leu-trp-his-ade* plates, were selected and expanded on new SD/*-leu-trp-his-ade* plates. The cells were collected and homogenized in 200 µl mp1 solution from plasmid mini-prep kits (Nippon Genetics Europe GmbH) with 200 µl glass beads at a speed of 10 m/s for 30 seconds by using MagNA Lyser (Roche, Switzerland)). Plasmids were further extracted by using plasmid mini-prep kits (Nippon Genetics Europe GmbH) and used to re-transform *E.coli* XL1 Blue cells for amplification. The plasmids were extracted again from *E.coli* cells and used to transform the yeast cells. To estimate the false positive background, the transformed yeast cells were mated with the yeast cells carrying the empty bait plasmid. In addition, full-length ORFs of candidate genes were cloned into Y2H vectors and used to further verify both their false positive background and their interaction with ErCry4. Although a diploid will be finally formed by two haploids cell, it seemed that prior to mating, the expression of two Y2H plasmids can vary in two haploid strains (see also Figure S1B).

### Immunoblotting

Yeast cells were grown to an OD_546_ of 0.6 or the concentration of 1×10^7^cells/ml and collected by centrifugation. A volume of 2x sodium dodecyl sulphate (SDS) sample buffer equivalent to the cell pellets was added and two volume of glass beads equivalent to the cell pellets were also added. The cells were disrupted by MagNA Lyser (Roche, Switzerland) followed by centrifugation. The supernatants were transferred to a new tube and further analysed by SDS gel electrophoresis (SDS-PAGE) and immuno detection. Composition of sample buffer, performance of SDS-PAGE and immune blotting is described elsewhere^26^. Proteins expressed from pDHB1 and pGBKT7-SfiI were detected by anti-LexA (1:500, clone E-7, sc-365999, Santa Cruz Biotechnology) and anti-myc (1:500, clone 9E10, sc-40, Santa Cruz Biotechnology) antibodies, respectively. Proteins expressed from pPRN3 and pGADT7-SfiI were detected by anti-HA antibodies (1:500, clone 3F10, Helmholtz Zentrum München, German Research Center for Environmental Health, Institute for Diabetes and Obesity, Monoclonal Antibody Core Facility).

## Supplementary information

Supplementary Information.
