# Supplementary material for: Protein-protein interaction of the putative magnetoreceptor cryptochrome 4 expressed in the avian retina
Source: Sci Rep. 2020 Apr 30;10:7364. doi: 10.1038/s41598-020-64429-y (PMC7193638; doi:10.1038/s41598-020-64429-y)
Supplement: Supplementary file 1 — Supplementary Information. [file 41598_2020_64429_MOESM1_ESM.pdf]

# Supplementary information

# Protein-protein interaction of the putative magnetoreceptor cryptochrome expressed in the avian retina

Haijia Wu[1], Alexander Scholten[1], Angelika Einwich[2], Henrik Mouritsen[2] and Karl-Wilhelm Koch[1]

[1]Department of Neuroscience, Division of Biochemistry, University of Oldenburg, D-26111 Oldenburg, Germany; [2]Department of Biology and Environmental Sciences, Neurosensorics/Animal Navigation, University of Oldenburg, D-26111 Oldenburg, Germany

### Comparison of the two Y2H-systems

The split-ubiquitin system (supplementary Figure S1A, lower panel) employs the protein ubiquitin that is separated in a C-terminal moiety (Cub) and a mutated N-terminal moiety (NubG). Both moieties are fused to either the bait or the prey. They can only re-join to a whole ubiquitin, when bait and prey interact. Reassembled ubiquitin could then be recognised by a specific protease, which releases the transcription factor LexA-VP16 that, in turn, triggers the expression of the reporter genes[52]. A further difference of the split-ubiquitin system compared with the UAS-GAL4 system lies in the transformation of yeast cells. In the UAS-GAL4 system, bait and prey vectors co-exist in one cell, which is achieved by formation of a diploid cell through mating two haploid cells, which are transformed by the bait or the prey vector, respectively. In the split-ubiquitin system, both bait and prey vectors are used in a double-transformation of a single yeast strain (supplementary Figure S1B). In both Y2H systems, specific growing on selective plates requires the interaction of the bait and prey proteins and thus indicates the successful strains of transformation or mating. Vector maps are shown in

Figure S2. Table S1 summarizes the main differences of the materials used in the two Y2H systems.

**Figure S1**

**A**

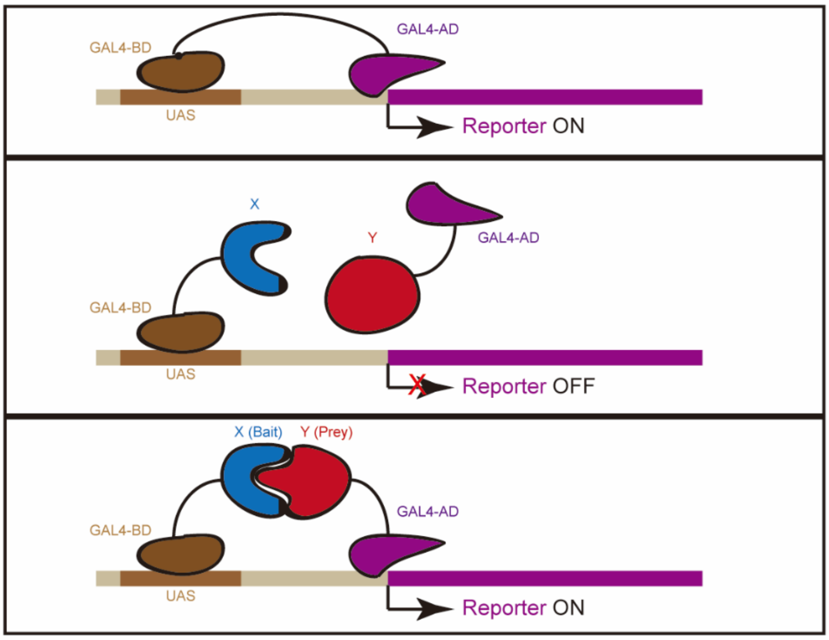


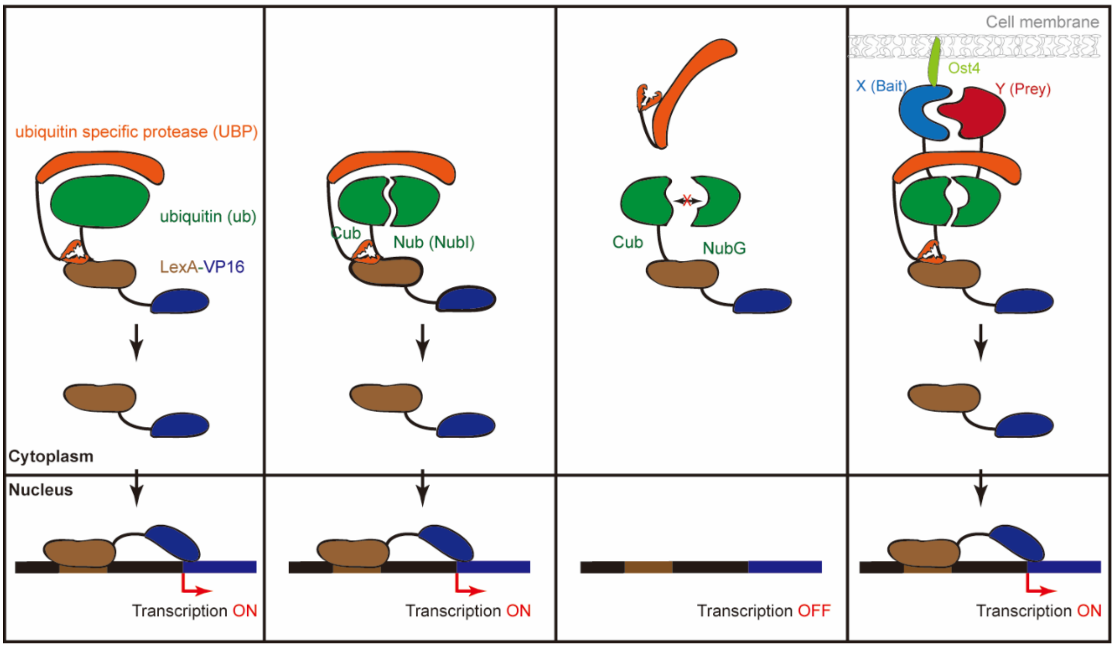

**B**

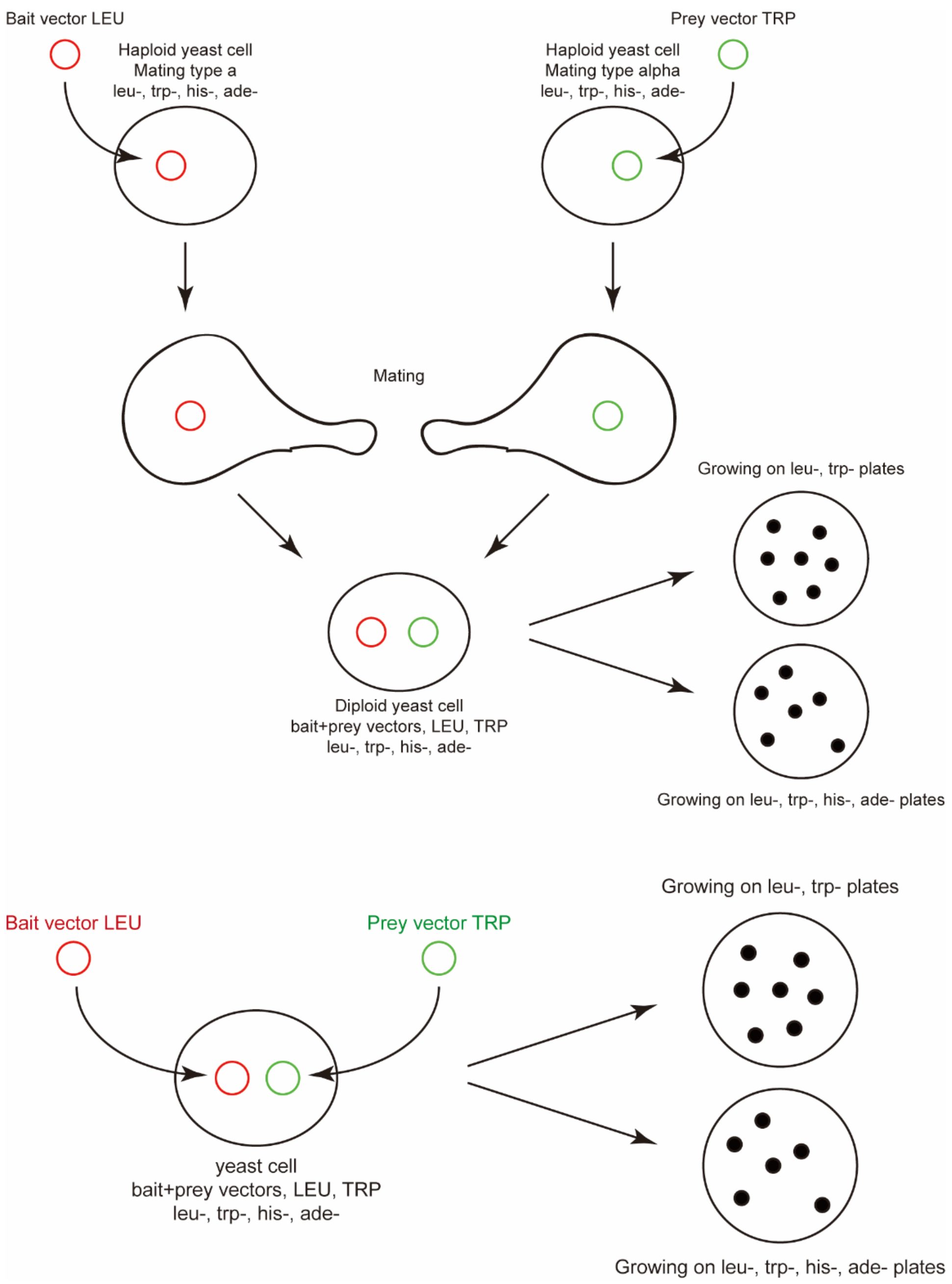


**Figure S1.** Design principles of the Y2H screening. (A) Comparison of the two Y2H systems used in the present study, the UAS-GAL4 system (upper part) and the split ubiquitin system (lower part). Events are shown that lead to turning on the expression of a reporter gene. (B) Transformation of yeast cells. In the UAS-GAL4 system, bait and prey vectors co-exist in one cell, which is achieved by formation of a diploid cell through mating two haploid cells (upper panel in B). In the split-ubiquitin system, both bait and prey vectors are used in a double-transformation of a single yeast strain (lower panel in B). In both Y2H systems, specific

growing on selective plates requires the interaction of the bait and prey proteins and thus indicates the successful strains of transformation or mating.

**Figure S2**

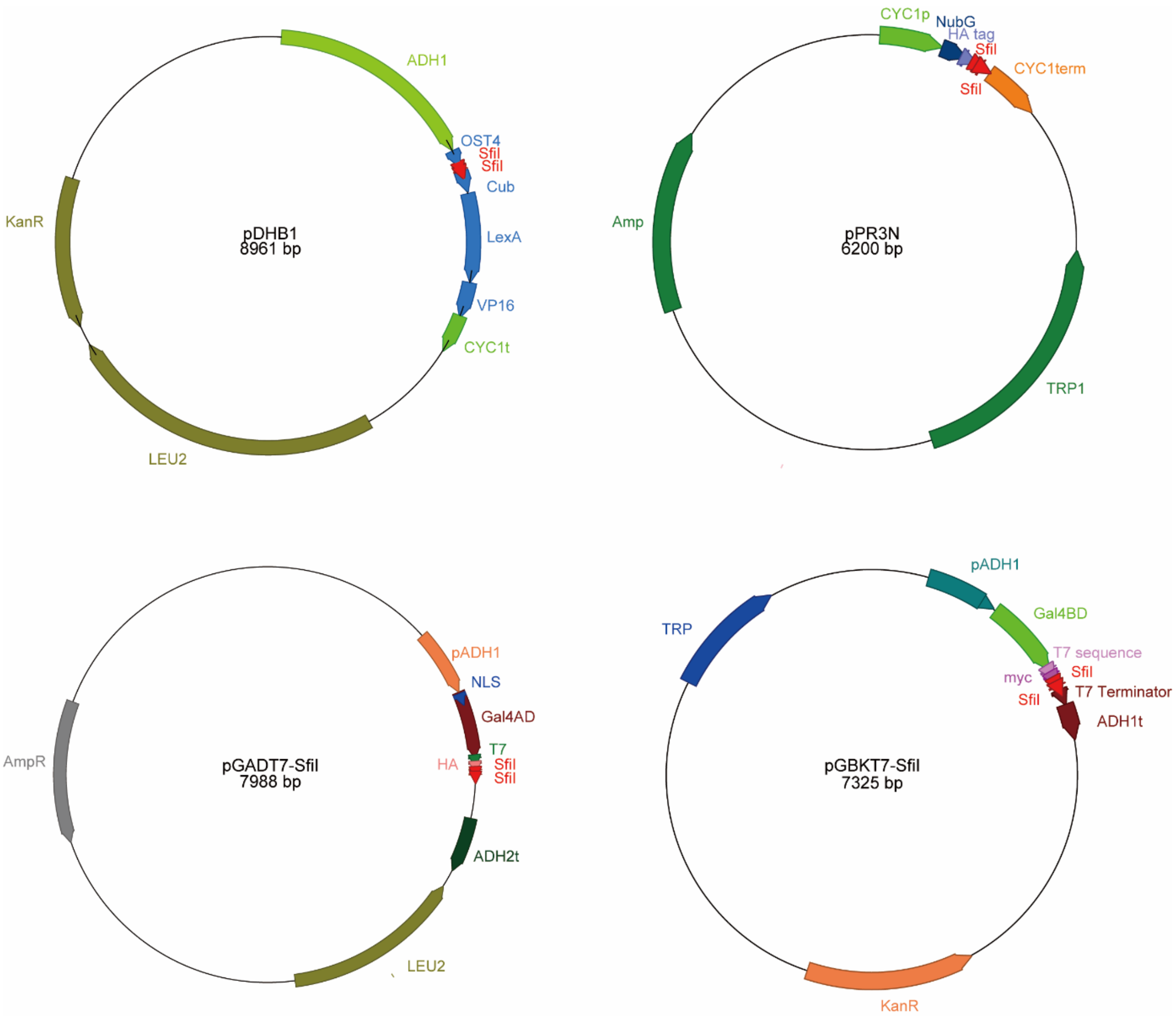


**Figure S2:** Vector maps used in Y2H screening.

**Figure S3**

**A**

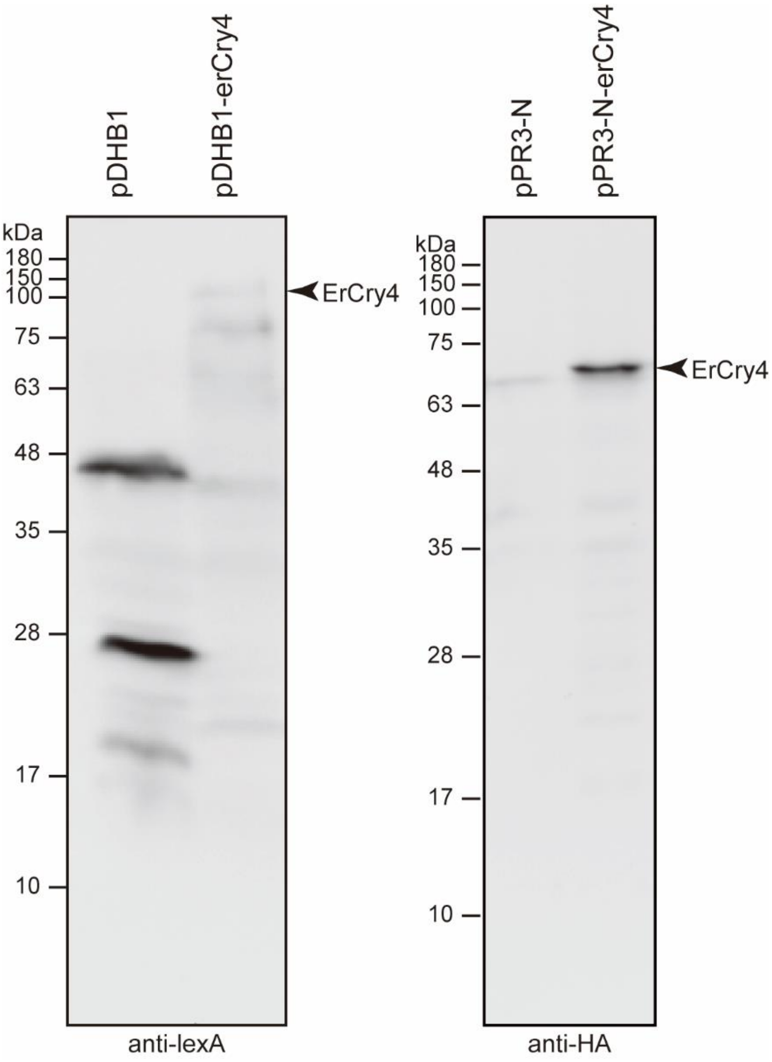


**Figure S3A:** Testing the usability of the split-ubiquitin Y2H system in screening for ErCry4 interaction partners. (A) Western Blots demonstrate the expression of ErCry4 (arrow) using the pDHB1 (left panel) or the pPR3-N vector (right panel). The lexA antibody (1:500) was used to detect pDHB1 expressed proteins, the HA antibody (1:500) was used to detect pPR3-N proteins. Strong background staining was seen in the empty control vector with the anti-lexA antibody (left lanes in the left panel), but specific staining of ErCry4 (arrow) was very weak (right lane in left panel). Expression of ErCry4 using the pPR3-N vector was detected by the anti-HA antibody (right lane in right panel).

**B**

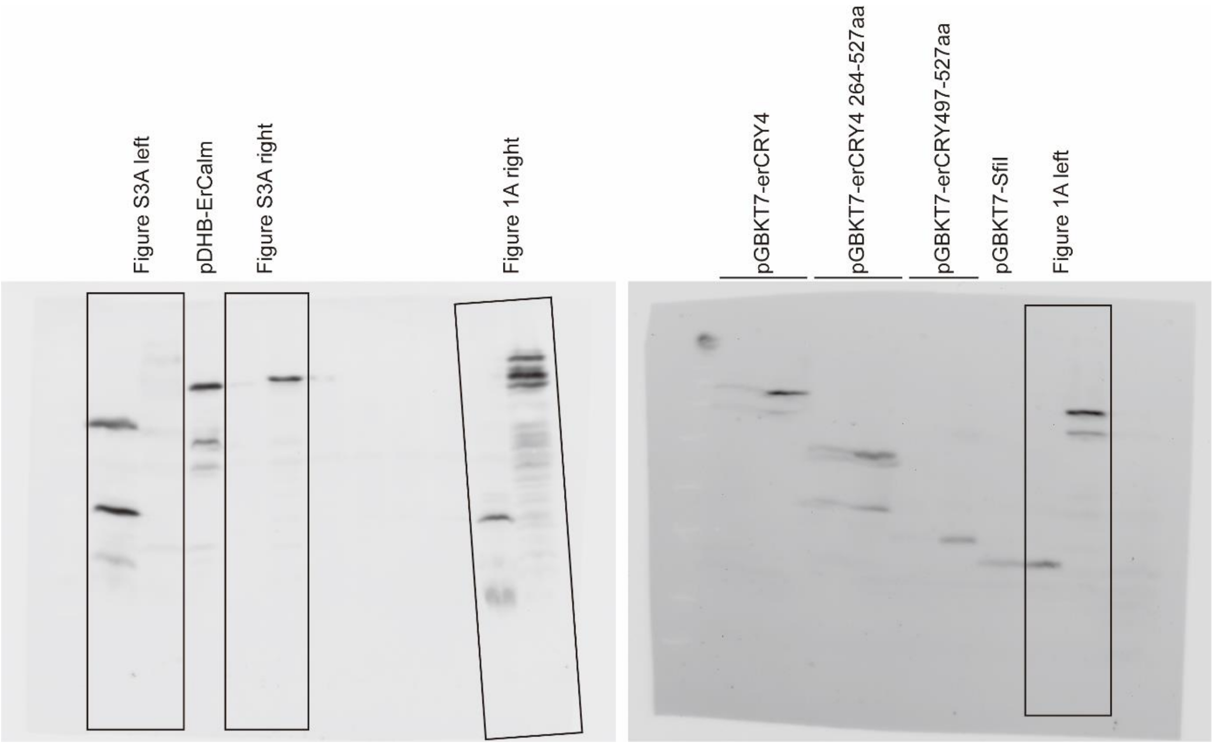


**Figure S3B:** Original blots to Figure 1A and Figure S3 (see above). The blots were cut and assembled as indicated. Lanes out of frame show experimental tests not reported in the main text.

**C**

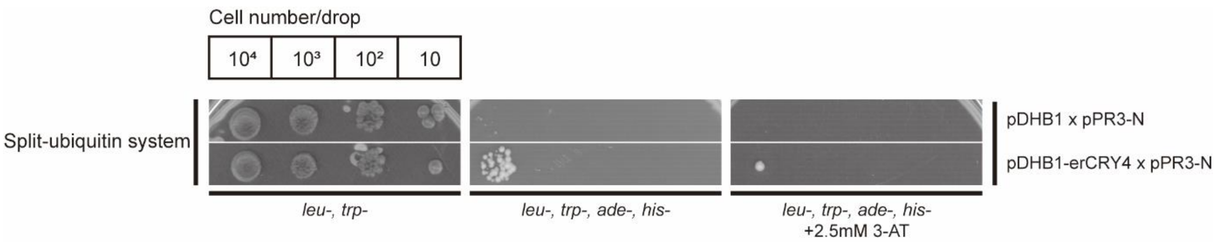


**Figure S3C:** Serial drop tests were used to verify the background interaction of ErCry4 in the split-ubiquitin system. Details are described in the main text in the legend to Figure 1B.

**Figure S4:**

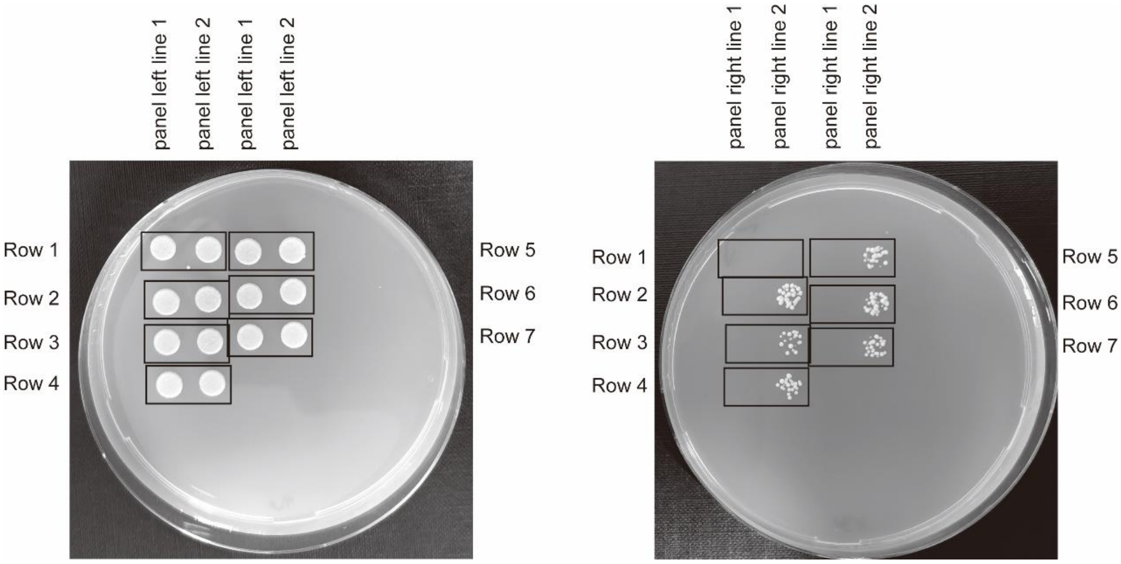


**Figure S4A:** Original figures of the full plates to Figure 4A (rows and lines in left and right panel as indicated). The figures of the plates were cut and assembled as framed.

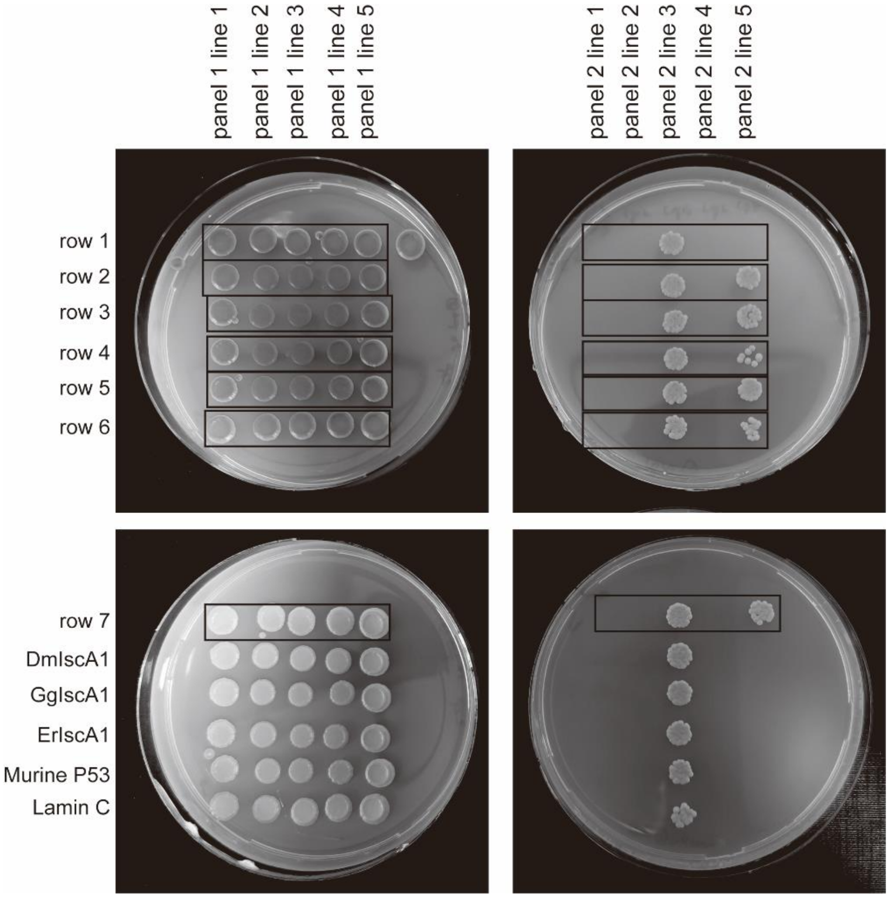


**Figure S4B:** Original figures of the full plates to Figure 4B (row 1-6, upper part and row 7, lower part). Rows were cut and assembled as indicated. Parts, which are not framed relate to preliminary tests not reported in the manuscript. They show tests that gave inconclusive results of interaction studies between Cry forms with DmIscA1, GgIscA1, ErIscA1, Murine P53 and Lamin C.

**Figure S5**

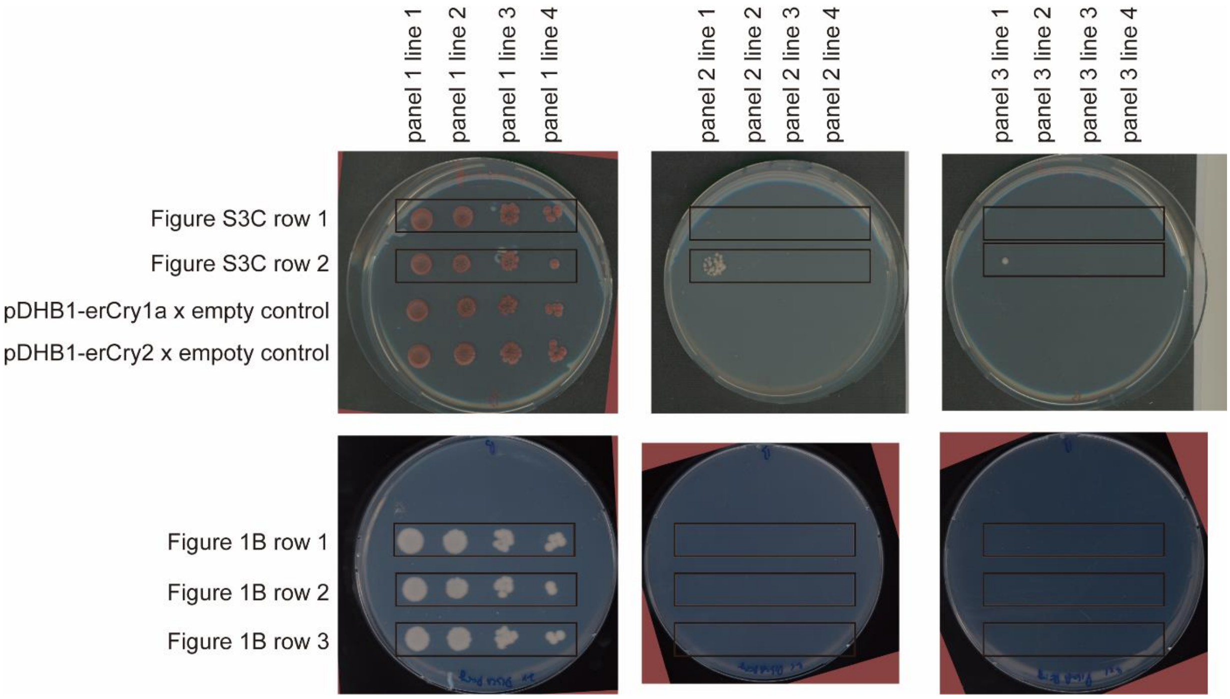


**Figure S5:** Original figures of the full plates to Figure S3C (row 1 and 2) and Figure 1B (row 1-3). The figures of the plates were cut and assembled as indicated. The non-framed part shows background tests of ErCry1a and ErCry2 using the split-ubiquitin-system that was not further used in the study (see also main text).

**Figure S6**

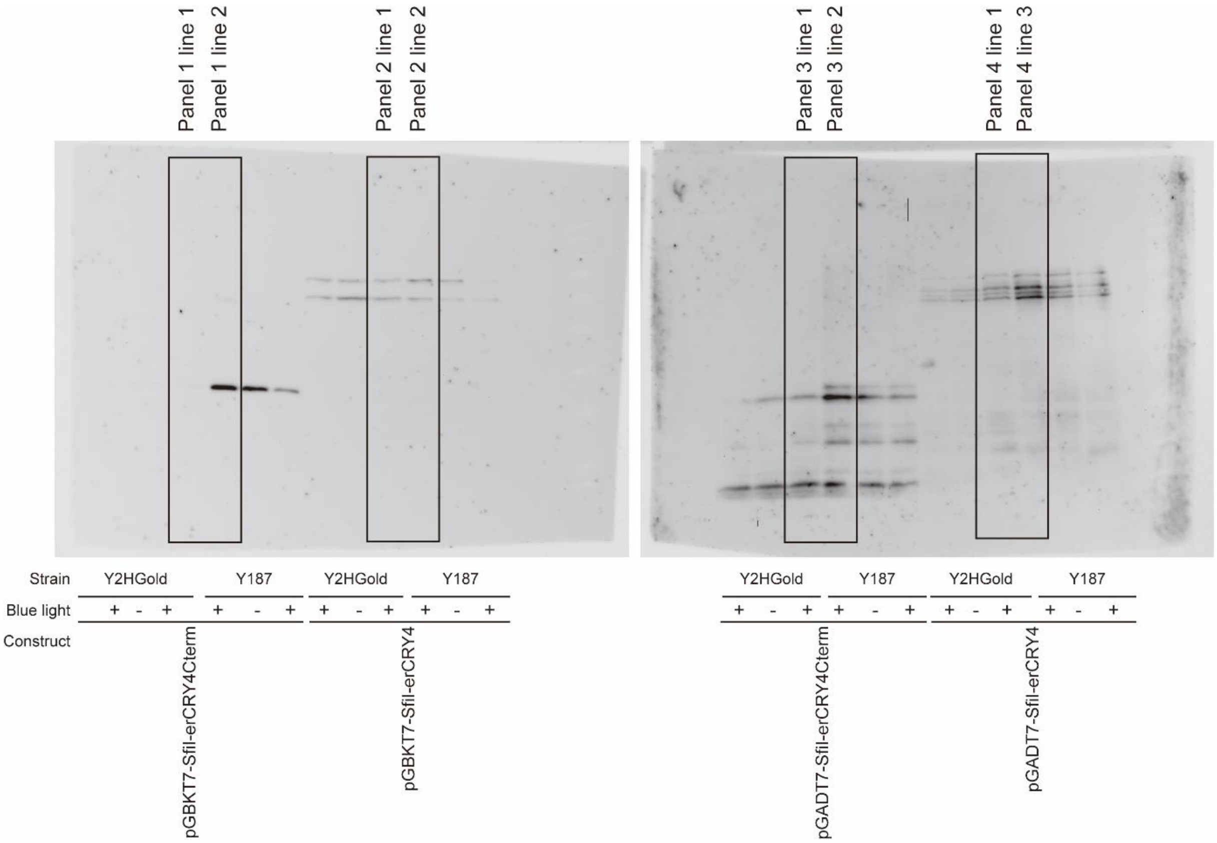


**Figure S6:** Original blots to Figure 5 (left). The blots were cut and assembled as indicated. Presence or absence of blue light is indicated.

**Figure S7**

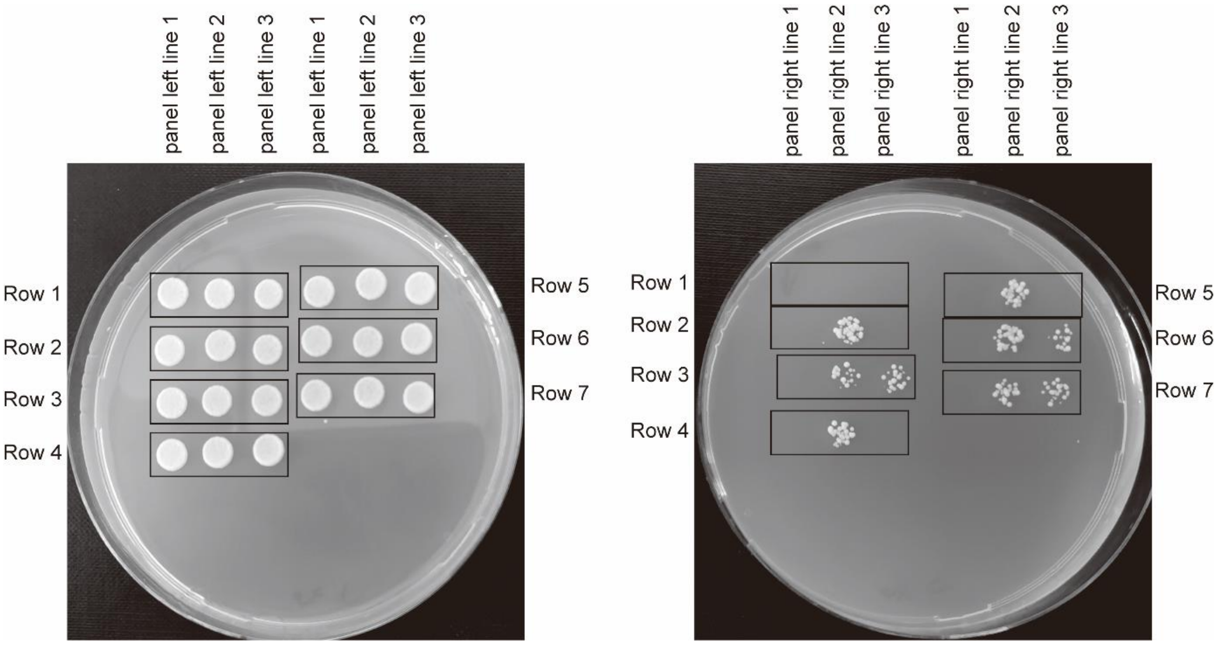


**Figure S7:** Original figures of the full plates to Figure 5 (right). The figures of the plates were cut and assembled as framed.

**Figure S8**

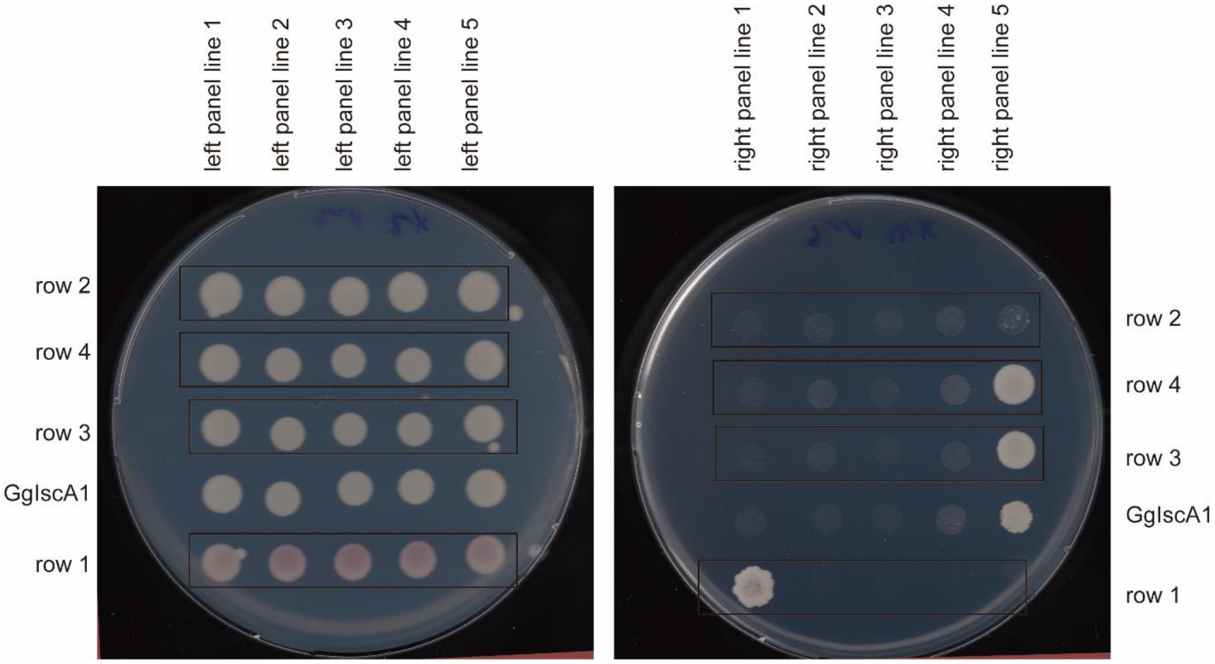


**Figure S8:** Original figures of the full plates to Figure 6A. The figures of the plates were cut and assembled as framed. The non-framed row shows interactions tests of GgIscA1 and proteins indicated in Figure 6A. Apparent interaction of GgIscA1 with DmIscA1 (spot in right panel, line 5) was inconclusive and not confirmed so far.

**Figure S9**

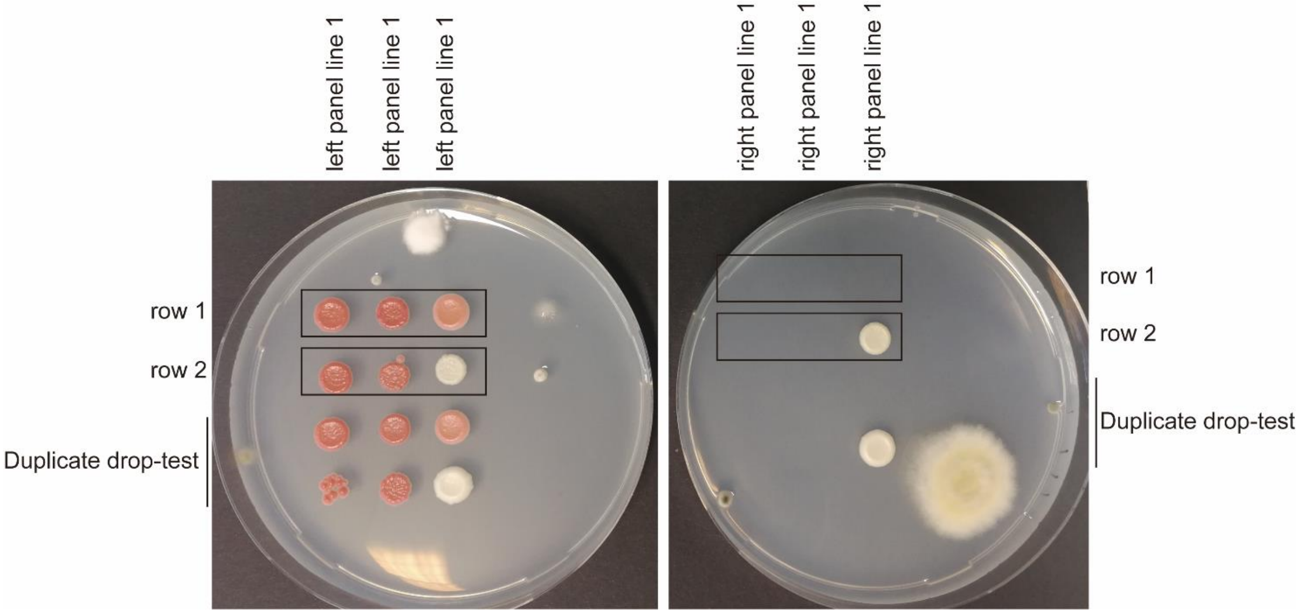


**Figure S9:** Original figures of the full plates to Figure 6B. The figures of the plates were cut and assembled as framed.

**Table S1.** Comparison of the two Y2H systems used in the screening approach. Details of the genetic properties and specificities concerning the yeast strains and plasmids

| | Split-ubiquitin system | UAS-GAL4 system |
|---|---|---|
| Yeast strains | **NMY31**<br>*his3Δ, trp1Δ, leu2Δ, ade2Δ, (LexAop)x4-HIS3, (LexAop)x4-ADE2* | **Y2Hgold**<br>MATa *his3Δ, trp1Δ, leu2Δ, ade2Δ GAL1uas-GAL1tata-HIS3 GAL2uas-GAL2tata-ADE2* |
| | | **Y187**<br>MATα *his3Δ, trp1Δ, leu2Δ, ade2Δ* |
| Plasmids | **pDHB1**<br>*pADH1:OST4-SfiI-SfiI-Cub-LexA-VP16:CYC1t LEU2 KanR* | **pGBKT7-SfiI**<br>*pADH1:GAL4BD-pT7-myc-SfiI-SfiI:T7t:ADH1t TRP1 KanR 2μ* |
| | **pPR3-N**<br>*pCYC1:NubG-HA-SfiI-SfiI:CYC1t TRP1 AmpR* | **pGADT7-SfiI**<br>*pADH1:NLS-GAL4AD-pT7-HA-SfiI-SfiI:ADH1t LEU2 AmpR 2μ* |

**Table S2. Primer sequences used in the present study**

| Number | Primer | Sequence |
|---|---|---|
| 227 | ErCry4 fwd | TCGCGGCCATTACGGCCATGCTGCATCGCACCATTCA |
| 228 | ErCry4 rev | TCGCGGCCGAGGCGGCCAAGTCTGTCGTTCGGGCTGC |
| 639 | ErCry4 rev | GAGGCCGAGGCGGCCGTTATTCTGTTGTTCGGGCCAC |
| 465 | ErCry4c fwd | GCGGCCATTACGGCCATGGTGAACCACAAGGAAGCCAG |
| 226 | GgCry4 fwd | TCGCGGCCATTACGGCCATGCGGCACCGCACC |
| 225 | GgCry4 rev | TCGCGGCCGAGGCGGCCAAGGTTGGCTCTGTCATCCTG |
| 369 | GgCry4 rev | GAGAGGCCGAGGCGGCCGTTAGGTTGGCTCTGTCATCCTGG |
| 502 | DmCry fwd | GCGGCCATTACGGCCATGGCCACGCGAGGG |
| 503 | DmCry rev | ATGGCCGAGGCGGCCAAAACCACCACGTCGGCC |
| 504 | DmCry rev | GAGGCCGAGGCGGCCGTCAAACCACCACGTCGGC |
| 499 | DmIscA1 fwd | GTGGCCATTACGGCCATGGCGACACGTGTGGTG |
| 500 | DmIscA1 rev | ATGGCCGAGGCGGCCAACATGCTGAACGATTCGCCG |
| 501 | DmIscA1 rev | GAGGCCGAGGCGGCCGTTACATGCTGAACGATTCGCCG |

| | | |
|---|---|---|
| 271 | ErIscA1 fwd | GAGTGGCCATTACGGCCATGGCATCGTCGGTGGTG |
| 272 | ErIscA1 rev | GAGGGGCCGAGGCGGCCGTCAAATGTTAAAGCTTTCTCCACAGC |
| 389 | RGR fwd | GTGGCCATTACGGCCATGGTCACTTCACATCCTCTCCC |
| 390 | RGR rev | GGGGCCGAGGCGGCCGTTACTTAGTTTTGTTATCAACCTCTGCTTTCTC |
| 541 | GNAT2 fwd | GTGGCCATTACGGCCATGGGGAGCGGGGC |
| 542 | GNAT2 rev | GAGGCCGAGGCGGCCGTCAGAAGAGCCCGCAGTC |
| 543 | GNG10 fwd | GTGGCCATTACGGCCATGTCGTCGGCCGGC |
| 544 | GNG10 rev | GAGGCCGAGGCGGCCGTCAGAGCAGAGTGCAGGATCT |
| 545 | RBP1 fwd | GTGGCCATTACGGCCATGCCTGCAGATTTCAATGGTTACT |
| 546 | RBP1 rev | GAGGCCGAGGCGGCCGTCACTGTACCTTCTTAAAGACCTGCT |
| 603 | LWO fwd | GCGGCCATTACGGCCATGGCGACGGGCGTTT |
| 605 | LWO rev | GAGGCCGAGGCGGCCGCTAGGCGGGTGACCCGG |
| 693 | KCNV2 fwd | GTGGCCATTACGGCCATGTTGCAGTTTAACAGGCAGC |
| 694 | KCNV2 rev | GAGGCCGAGGCGGCCGTCATTGCTGTGATGGAGGGTG |
